# Pricing strategy of multi-oligopoly airlines based on service quality

**DOI:** 10.1371/journal.pone.0216651

**Published:** 2019-06-17

**Authors:** Hang Zhou, Shikang Zhou

**Affiliations:** College of Civil Aviation, Nanjing University of Aeronautics and Astronautics, Nanjing, Jiangsu, China; Shandong University of Science and Technology, CHINA

## Abstract

In recent years, with the rapid development of China's air transport industry and the change in market consumption structure, service quality has become one of the important factors affecting airline revenue. How to formulate a reasonable pricing strategy and maintain competitiveness in the fierce market competition has become an urgent problem for airlines. First, the impact factor of service quality level in the traditional pricing model is introduced and a static price competition model for multi-oligopoly airlines based on service quality is established in this paper. And then, a dynamic pricing model based on service quality of the multi-oligopoly airlines is established. The model incorporates the weight factor of service quality impact, which is used to indicate the weight of the service quality level in the process of airline dynamic pricing. The research results show that the service quality level of airlines has an indispensable influence on its development. Airlines should improve service quality as soon as possible to enhance market competitiveness and achieve sustainable development.

## 1. Introduction

Service quality refers to the extent to which a service can meet the needs of the serviced person.

Traditionally, China’s four airlines have strong control in the air transport market. But with the rapid development of the air transport industry, some newly developing airlines have gradually carved up market share by actively seeking differentiated competitive advantages. From the perspective of operation, the basic functions provided to passengers by airlines on the same route are consistent, and the competition among these airlines is based on the air route. Especially on the main routes, 4 or more airlines on 50% of the routes are snatching marketing resources, there is fierce competition among airlines [1]. In general, the air transport market in China presents a multi-oligopolistic pattern with fierce market competition among airlines. How to maintain the advantages and enhance the competitiveness of the market has become an urgent problem that the airline needs to solve.

With the development of the economic, the consumption structure of the public is moving toward the emphasis on ‘quality’, especially in traveling. The service quality has become one of the important factors affecting the ticket revenue, and it is the best way for aviation-related enterprises to increase added value and implement differentiated development strategies [2]. The survey shows that the proportion of tourists and business passengers accounted for more than 65% in China’s air transport market. The higher the service quality of airline, the higher the satisfaction of these passengers and the higher loyalty, and the market share of the airline can be increased. In short, high-quality service has become the core competitiveness of airline to improve the revenue and achieve sustainable development [3]. So it is necessary to construct a pricing model based on service quality to study the effect of service quality on airline revenue management (RM).

## 2. Related researches

Before the gradual opening of air traffic rights, the method of setting the price of the ticket is uniform in the International. At this time, the research on airline revenue management was mainly based on single routes, and the focus was on overselling. Mumbower S studied factors that affect passengers' purchase of a premium class, including seat price, ticket purchase time, number of passengers, and load factor and based on the research results, made substantial suggestions for airlines to increase revenue [4]. Aguirregabiria, Victor and others based on dynamic competition theory [5], studied the influence of airlines' demand distribution, cost and policy factors on the radial aviation network in the US market.

In the study of airline ticket price strategy, some scholars try to find out the nature of market competition and propose a reasonable strategy for airlines' competitive advantage. Zhang Yahua studied the two problems of price wars and price alliances in the air transport market. Research shows that the price war is cyclical, and in the case of a market downturn, price alliances are more likely to occur [6]. Dan Zhang focused on the dynamic pricing problem between multiple alternative flights, developed the Markov pricing model, and proposed a heuristic algorithm for summarizing ideas [7]. Moreno-Izquierdo L seeks to analyze the effect that the internet has on the price strategy of low cost airlines. The study showed that the most significant factors on pricing strategies were the number of rivals, the behavior of demand and costs [8]. Xia HS constructed a duopoly competition model of airlines based on different competitive strategy and rational, controlled and analyzed the model, they provided some suggestions for the price regulation for the airline market [9]. Hu Rong, etc. built a dynamic pricing model of three oligopoly airlines. They studied the model chaos control and proposed two methods for delaying the feedback of the model [10]. Song JQ compares and analyzes the fare strategies under the competitive characteristics of different air transport markets, and combines the market characteristics of China to introduce the characteristic variables of high-speed rail competition in fare analysis [11].

Since the 1970s, scholars have conducted a lot of research on the relevant aspects of service quality and achieved fruitful results. In 2016, Etemadsajadi et al. studied the impact of pre- and in-flight airline service quality on passenger satisfaction and loyalty, reflecting the importance of airline pre-flight service quality [12]. In 2012, Yang K C et al. used a simple structural equation model to analyze the relationship between the service quality of a low-cost airline, the company's image, and the behavioral intentions of passengers. The research results show that higher service quality has a positive impact on passengers' behavior intentions and company image [13]. In 2007, Abdullah K et al. used the SERVQUAL method to evaluate the perceptions of Malaysian customers on their airline service levels. The results show that the three aspects of service empathy, reliability and tangibility are usually the most concerned by passengers [14]. Li Ling used the method of analytic hierarchy process (AHP) and GRAP to quantitatively evaluate the competitiveness of China’s the three major airlines and international famous airlines, it is concluded that China's airlines need to be upgraded in terms of operating scale, service quality and production efficiency [15]. Zhang zan adopts a game theoretical approach to examine the impact of free offering on the competition between two firms in the presence of network effects, and it is shown that when a firm’s core product has a sufficient advantage in product quality, it is better for this firm to sell the bundle but for the other to use free strategy [16].

The existing researches on airline revenue management (RM) pay little attention to the impact of service quality on the competition strategy, and most of the study of the pricing strategy is based on the built of duopoly or three-oligopoly model. The traditional duopoly and three-oligopoly competition models are not suitable for the current market characteristics, due to the increasingly fierce competition in the air transport market. Thus, this paper built a pricing model of multi-oligopoly airlines, and the factor of service quality is involved in this model. The influence on market demand, revenue, and competition by the service quality level is analyzed so that the more reasonable development strategy is provided for the airline to achieve sustainable development.

## 3. Model and methods

Before constructing the service-quality-based multi-oligopoly airlines pricing model, this paper firstly needs to set some assumptions: 1) in order to focus on the impact of service quality on airline competition strategies, this paper only considers the impact of passenger ticket price and service quality on the market demand; 2) this paper assumes that the market demand of airlines is affected not only by the service quality and ticket price but also by these two factors of their rivals. The higher of service quality level its own and the ticket price of its rivals, the lower of market demand; the higher of ticket price of its own and the service quality level of its rivals, the lower the market demand of airline, it is in keeping with public awareness; 3) in order ensure the solvability and uniqueness of the solution of the model, this paper also assumes that the demand function is continuously differentiable, bounded and the inverse function exists.

### 3.1 Market demand function

In fact, a service provider can appropriately reduce the price to compensate for the loss of market demand caused by a low level of service quality or improve the service quality to compensate the loss of market demand caused by the high price, so as to achieve the stability of market demand. Based on the above, the service quality-based market demand function can be set as follows:

$$D_{i}=a_{i}-e_{i}p_{i}+f_{i}s_{i}+d_{i}p_{j}-g_{i}s_{j}$$ (3-1)

*D*_*i*_ is the market demand for *airline*_*i*_. *a*_*i*_ is the basic market demand, that is the original market share of *airline*_*i*_ without considering the factors of the ticket price and service quality, and it is depended on the degree of passengers’ acceptation to such factors as air route, time and brand. *e*_*i*_*p*_*i*_ represents the impact of ticket price *p*_*j*_ of *airline*_*i*_ on the market demand, *f*_*i*_*s*_*i*_ is the effort of *airline*_*i*_ to improve the market demand. *p*_*i*_ is the ticket price of *airline*_*i*_, *e*_*i*_ is the sensitivity coefficient of the ticket price to market demand, *s*_*i*_ is the service quality level of *airline*_*i*_, *f*_*i*_ is the sensitivity coefficient of service quality to market demand. *d*_*i*_*p*_*j*_ represents the impact of the ticket price of rival *airline*_*j*_ on market demand *D*_*i*_, *g*_*i*_*s*_*j*_ represents the impact of service quality of rival *airline*_*j*_ on market demand of *airline*_*i*_. *d*_*i*_ is the sensitivity coefficient of ticket price *p*_*i*_ of rival *airline*_*j*_ to the market demand of *airline*_*i*_, and *g*_*i*_ is the sensitivity coefficient of service quality *s*_*j*_ of rival *airline*_*j*_ to the market demand of *airline*_*i*_.

Further, the market demand function can be extended to a more general form. When there are multi-oligopoly airlines participating in market competition, the market demand function can be seen as following:

$$D_{i}=a_{i}-e_{i}p_{i}+f_{i}s_{i}+d_{i}\underset{j\neq i}{\sum }p_{j}-g_{i}\underset{j\neq i}{\sum }s_{j}$$ (3-2)

*d*_*i*_ is the joint sensitivity coefficient of ticket price *p*_*j*_ of rival airlines to the market demand of *airline*_*i*_, which means the oligopoly airlines excludes their own factors, the combined effect of competitors' fares on *airline*_*i*_’s market demand. *g*_*i*_ is the joint sensitivity coefficient of service quality *s*_*j*_ of rival airlines to the market demand of *airline*_*i*_, which means the oligopoly airlines excludes their own factors, the combined impact of competitors' quality of service on *airline*_*i*_’s market demand.

### 3.2 Variable cost function

**Fig 1:**
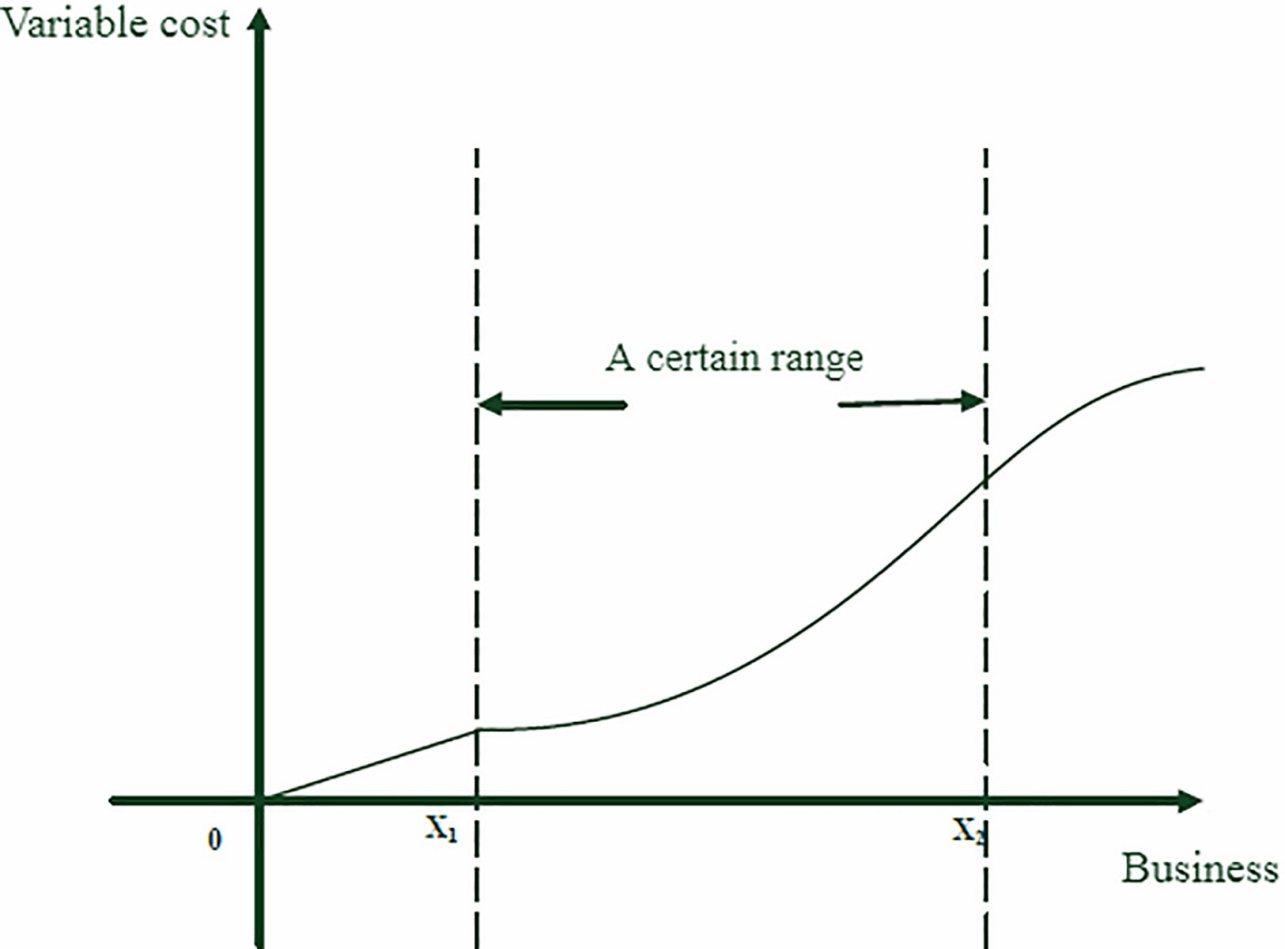
Variable cost function.

Actually, the airline should focus on the variable cost, especially considering the short-term competition, which may make competition strategy by the airline be more effective. The variable cost of airline mainly includes the fare incurred by various projects such as fuel, original materials, landing service, catering, reservation and so on. Variable cost refers to a cost item that changes with a change in business volume within a certain scale. Overall, the variable costs of airline operations grow as market demand grows. In the early stage of the growth of the business, the variable cost and business volume are linear. As the business continues to grow, the variable cost and the business are usually nonlinear. The relationship between business and variable cost is rough as shown in Fig 1.

The business volume can be seen as the market demand for the airline. For convenience, this paper stipulates the relationship between the variable cost of the airline and the market demand is quadratic function, and the function can be seen as following:

$$C_{it}=\theta_{i}D_{it}^{2}$$ (3-3)

*C*_*it*_ is the variable cost of *airline*_*i*_ at period t and *C*_*it*_>0, *D*_*it*_ is the market demand of *airline*_*i*_ at period t; *θ*_*i*_ is the growth coefficient and *θ*_*i*_>0, which indicates the degree of change in variable costs as market demand grows.

### 3.3 Service quality-based static pricing model of multi-oligopoly airlines

In reality, a service provider can compensate for the loss of demand caused by low-level service quality level by appropriately lowering the price, or balance the market demand caused by high price by improving the service quality level, and make its own market demand. It remains stable, but it cannot guarantee that the needs of competitors in other markets will not change. Therefore, when establishing the airline's four demand models, not only must consider the impact of its own fare and service quality level, but also consider the competitor's service level and fare. This model is based on the four oligarchic airlines. The current status of competition in China's air transport market will help Chinese airlines to formulate more reasonable pricing strategies.

This paper mainly focuses on the competition between four-oligarchic airlines, and firstly constructs the service quality-based marker demand function of four-oligopoly.

$$\left\{ \begin{array}{l} D_{1}=a_{1}-e_{1}p_{1}+f_{1}s_{1}+d_{1}(p_{2}+p_{3}+p_{4})-g_{1}(s_{2}+s_{3}+s_{4})\\ D_{2}=a_{2}-e_{2}p_{2}+f_{2}s_{2}+d_{2}(p_{1}+p_{3}+p_{4})-g_{2}(s_{1}+s_{3}+s_{4})\\ D_{3}=a_{3}-e_{3}p_{3}+f_{3}s_{3}+d_{3}(p_{1}+p_{2}+p_{4})-g_{3}(s_{1}+s_{2}+s_{4})\\ D_{4}=a_{4}-e_{4}p_{4}+f_{4}s_{4}+d_{4}(p_{1}+p_{2}+p_{3})-g_{4}(s_{1}+s_{2}+s_{3}) \end{array}\right.$$ (3-4)

The revenue function of *airline*_*i*_ is *π*_*i*_ = *p*_*i*_*D*_*i*_−*C*_*i*_. The aim of the research on pricing strategy is the maximization of revenue. Assume that the maximum revenue function is differentiable, the first order of maximum revenue is $\frac{\partial \pi (x)}{\partial x_{i}}=\frac{\partial C(x)}{\partial x_{i}}$. Thus, the marginal profit function of oligarchic airline can be showed as:

$$\left\{ \begin{array}{l} \xi_{1}=\frac{\partial \pi_{1}}{\partial p_{1}}=a_{1}-{2e}_{1}p_{1}+f_{1}s_{1}+d_{1}(p_{2}+p_{3}+p_{4})-g_{1}(s_{2}+s_{3}+s_{4})\\ \xi_{2}=\frac{\partial \pi_{2}}{\partial p_{2}}=a_{2}-{2e}_{2}p_{2}+f_{2}s_{2}+d_{2}(p_{1}+p_{3}+p_{4})-g_{2}(s_{1}+s_{3}+s_{4})\\ \xi_{3}=\frac{\partial \pi_{3}}{\partial p_{3}}=a_{3}-{2e}_{3}p_{3}+f_{3}s_{3}+d_{3}(p_{1}+p_{2}+p_{4})-g_{3}(s_{1}+s_{2}+s_{4})\\ \xi_{4}=\frac{\partial \pi_{4}}{\partial p_{4}}=a_{4}-{2e}_{4}p_{4}+f_{4}s_{4}+d_{4}(p_{1}+p_{2}+p_{3})-g_{4}(s_{1}+s_{2}+s_{3}) \end{array}\right.$$ (3-5)

Further, the equilibrium ticket price of each oligarchic airline can be obtained, namely $p^{*}=(p_{1}^{*},p_{2}^{*},p_{3}^{*},p_{4}^{*})$, which is the Nash optimal solution of this competition system.

### 3.4 Service quality-based dynamic pricing model of multi-oligopoly airlines

In the actual situation, the information in the market always changes over time, and airline is usually unable to obtain comprehensive market information due to the limited rationality of decision. The pricing strategy obtained from the static model is based on the theory of complete rational decision, which is an ideal model and leads to the incomprehensive of results. This paper further considers the pricing strategy of airline form the perspective of multi-cycle game, so as to increase the scientificity and rationality of the results. Firstly, a dynamic market demand function of oligarchic airlines is established and analyses the influence of service quality on the market competition under the consideration of time period.

$$\left\{ \begin{array}{l} D_{1t}=a_{1}-e_{1}p_{1t}+f_{1}s_{1}+d_{1}(p_{2t}+p_{3t}+p_{4t})-g_{1}(s_{2}+s_{3}+s_{4})\\ D_{2t}=a_{2}-e_{2}p_{2t}+f_{2}s_{2}+d_{2}(p_{1t}+p_{3t}+p_{4t})-g_{2}(s_{1}+s_{3}+s_{4})\\ D_{3t}=a_{3}-e_{3}p_{3t}+f_{3}s_{3}+d_{3}(p_{1t}+p_{2t}+p_{4t})-g_{3}(s_{1}+s_{2}+s_{4})\\ D_{4t}=a_{4}-e_{4}p_{4t}+f_{4}s_{4}+d_{4}(p_{1t}+p_{2t}+p_{3t})-g_{4}(s_{1}+s_{2}+s_{3}) \end{array}\right.$$ (3-6)

*D*_*it*_(*i* = 1,2,3,4) is the market demand of each oligarchic airline at *peridod*_*t*_, *s*_*i*_ is the service quality of *airline*_*i*_, *p*_*it*_ is the ticket price of airline_*i*_ at the *peridod*_*t*_, and *a*_*i*_,*e*_*i*_,*f*_*i*_,*d*_*i*_,*g*_*i*_>0. The dynamic profit function of *airline*_*i*_ can be shown as:

$$\pi_{it}=p_{it}D_{it}-C_{it}=p_{it}(a_{i}-e_{i}p_{it}+f_{i}s_{i}+d_{i}\underset{j\neq i}{\sum }p_{jt}-g_{i}\underset{j\neq i}{\sum }s_{j})-\theta_{i}D_{it}^{2}/2$$ (3-7)

Further, the marginal profit function *ξ*_*it*_ of *airline*_*i*_ at the *peridod*_*t*_ can be shown as:

$$\frac{\partial \pi_{it}}{\partial p_{it}}=\xi_{it}=D_{it}+p_{it}\frac{\partial D_{it}}{\partial p_{it}}-2\theta_{i}D_{it}\frac{\partial D_{it}}{\partial p_{it}}=(1+2\theta_{i}e_{i})D_{it}-e_{i}p_{it}$$ (3-8)

Thus, the dynamic marginal profit of each oligarchic airline can be specified as:

$$\left\{ \begin{array}{l} \xi_{1t}=(1+2\theta_{1}e_{1})(a_{1}+f_{1}s_{1}-g_{1}(s_{2}+s_{3}+s_{4}))-(2e_{1}+2\theta_{1}e_{1}^{2})p_{1t}+(d_{1}+2\theta_{1}e_{1}d_{1})(p_{2t}+p_{3t}+p_{4t})\\ \xi_{2t}=(1+2\theta_{2}e_{2})(a_{2}+f_{2}s_{2}-g_{2}(s_{1}+s_{3}+s_{4}))-(2e_{2}+2\theta_{2}e_{2}^{2})p_{2t}+(d_{2}+2\theta_{2}e_{2}d_{2})(p_{1t}+p_{3t}+p_{4t})\\ \xi_{3t}=(1+2\theta_{3}e_{3})(a_{3}+f_{3}s_{3}-g_{3}(s_{1}+s_{2}+s_{4}))-(2e_{3}+2\theta_{3}e_{3}^{2})p_{3t}+(d_{3}+2\theta_{3}e_{3}d_{3})(p_{1t}+p_{2t}+p_{4t})\\ \xi_{4t}=(1+2\theta_{4}e_{4})(a_{4}+f_{4}s_{4}-g_{4}(s_{1}+s_{2}+s_{3}))-(2e_{4}+2\theta_{4}e_{4}^{2})p_{4t}+(d_{4}+2\theta_{4}e_{4}d_{4})(p_{1t}+p_{2t}+p_{3t}) \end{array}\right.$$ (3-9)

In order to increase the scientific and rationality of the results, it is assumed that the dynamic pricing decision is based on the combination of marginal profit, the ticket price and the service quality of last period. And the rate of price adjustment is influenced by the weighting factor of service quality. The dynamic pricing function is expressed as:

$$p_{i(t+1)}=p_{it}+(\beta_{i}+\alpha_{i})p_{it}\frac{\partial \pi_{it}}{\partial p_{it}}$$ (3-10)

$$p_{it}=(1-\beta_{i})p_{it}+\beta_{i}p_{i(t-1)}$$ (3-11)

(*β*_*i*_+*α*_*i*_) represents the adjustment rate of ticket price between the two adjacent periods, *α*_*i*_ is the macro adjustment rate of ticket price between the two adjacent periods, *β*_*i*_∈[0,1] is the weighting factor of service quality, and it means that with the improvement of quality of service levels, balanced fare airline also increased accordingly. Therefore, the higher the weight of the service quality level is considered in the next cycle, and the fare on behalf of the airline increases as the cycle increases. When *β*_*i*_ = 0, it means that the airline does not consider the factor of service quality to be priced; when *β*_*i*_ = 1, it means that the airline fully considers the quality of service of the previous cycle for the next stage of price setting, when *β*_*i*_∈[0,1], it indicates that the oligarchic airlines have a weighted influence on the price decision in the previous cycle when making price decisions.

Therefore, the dynamic pricing price competition model of multi-oligopoly airlines based on service quality is:

$$\left\{ \begin{array}{l} p_{1(t+1)}=p_{1t}+(\beta_{1}+\alpha_{1})p_{1t}[(1+2\theta_{1}e_{1})(a_{1}+f_{1}s_{1}-g_{1}(s_{2}+s_{3}+s_{4t}))-(2e_{1}+2\theta_{1}e_{1}^{2})((1-\beta_{1})p_{1t}+\beta_{1}p_{1(t-1)})\\ \;+(d_{1}+2\theta_{1}e_{1}d_{1})((1-\beta_{2})p_{2t}+\beta_{2}p_{2(t-1)}+(1-\beta_{3})p_{3t}+\beta_{3}p_{3(t-1)}+(1-\beta_{4})p_{4t}+\beta_{4}p_{4(t-1)})]\\ p_{2(t+1)}=p_{2t}+(\beta_{2}+\alpha_{2})p_{2t}[(1+2\theta_{2}e_{2})(a_{2}+f_{2}s_{2}-g_{2}(s_{1}+s_{3}+s_{4}))--(2e_{2}+2\theta_{2}e_{2}^{2})((1-\beta_{2})p_{2t}+\beta_{2}p_{2(t-1)})\\ \;+(d_{2}+2\theta_{2}e_{2}d_{2})((1-\beta_{1})p_{1t}+\beta_{1}p_{1(t-1)}+(1-\beta_{3})p_{3t}+\beta_{3}p_{3(t-1)}+(1-\beta_{4})p_{4t}+\beta_{4}p_{4(t-1)})]\\ p_{3(t+1)}=p_{3t}+(\beta_{3}+\alpha_{3})p_{3t}[(1+2\theta_{3}e_{3})(a_{3}+f_{3}s_{3}-g_{3}(s_{1}+s_{2}+s_{4}))-(2e_{3}+2\theta_{3}e_{3}^{2})((1-\beta_{3})p_{3t}+\beta_{3}p_{3(t-1)})\\ \;+(d_{3}+2\theta_{3}e_{3}d_{3})((1-\beta_{1})p_{1t}+\beta_{1}p_{1(t-1)}+(1-\beta_{2})p_{2t}+\beta_{2}p_{2(t-1)}+(1-\beta_{4})p_{4t}+\beta_{4}p_{4(t-1)})]\\ p_{4(t+1)}=p_{4t}+(\beta_{4}+\alpha_{4})p_{4t}[(1+2\theta_{4}e_{4})(a_{4}+f_{4}s_{4}-g_{4}(s_{1}+s_{2}+s_{3}))-(2e_{4}+2\theta_{4}e_{4}^{2})((1-\beta_{4})p_{4t}+\beta_{4}p_{4(t-1)})\\ \;+(d_{4}+2\theta_{4}e_{4}d_{4})((1-\beta_{1})p_{1t}+\beta_{1}p_{1(t-1)}+(1-\beta_{2})p_{2t}+\beta_{2}p_{2(t-1)}+(1-\beta_{3})p_{3t}+\beta_{3}p_{3(t-1)})] \end{array}\right.$$ (3-12)

The factor of service quality level influence weight is introduced in the dynamic pricing model, it can analyze the impact of service quality level influence weight on the competition cycle and the impact on the average profit of each oligarchic airline, help airlines make better decisions.

The factor of service quality level influence weight is introduced in the dynamic pricing model, it can analyze the impact of service quality level influence weight on the competition cycle and the impact on the average profit of each oligarchic airline, help airlines make better decisions.

Most of the previous literature only considered fixed costs, or a single cost factor, resulting in a pricing strategy that was not reasonable enough. In actual operations, the factors affecting the operating costs of airlines are multifaceted. The dynamic pricing model takes into account the variable cost factor, making the pricing strategy more practical.

## 4 Experiment and Analysis

### 4.1 The impact of service quality level on market demand

In order to help the airline to make a reasonable competition strategy to enhance market competitiveness and brand influence, this section mainly focuses on the relationship between the service quality level and market demand. The values of the four oligarchic airlines are assigned according to the evaluation results in the quarterly airline service evaluation report released by the Civil Aviation Passenger Service Evaluation (CAPSE). The range of service quality level is set as [1,10], which is divided into three grades: high, medium and low. It is called low level when the value of service quality level in the interval [1,3], and the interval [4,7] represents the intermediate level, and the interval [8,10] is the high level. Based on the market demand model, we analyze the market share of each airline in different service quality levels. The results can be seen in Table 1. We set the *s*_*i*_ with the same value in each level interval and obtain the market share.

**Table 1:** Market share of different airlines.

market demand ratio(%) service quality level interval	Hainan Airlines	Southern Airlines	Eastern Airlines	International aviation
[1,3]	30.2	23.7	25.4	20.7
[4,7]	26.9	19.8	25.3	28
[8,10]	15.2	21.6	27.6	35.3

**Fig 2:**
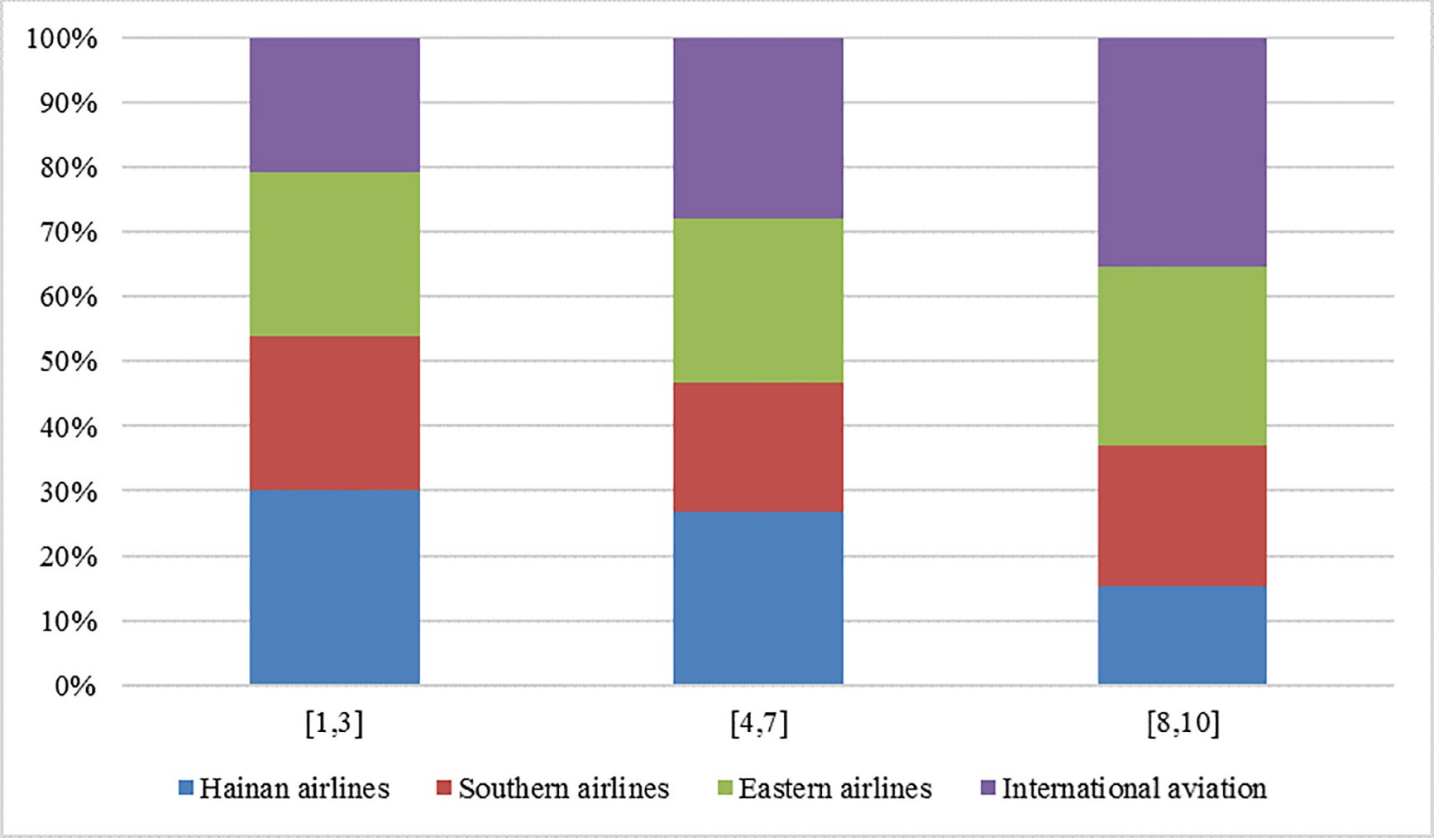
Bar chart of airline market share.

In order to facilitate comparison and analysis, the market share of each airline is shown in Fig 2.

As can be seen in Fig 2, at the interval of low service quality level, the market demand of Hainan airline accounted for the most, and with the increase of service quality level the market shares of a downward trend. While the market share of International aviation is relatively low, and with the increase of service quality level, the proportion of market demand increases. Actually, compared to the other three oligarchic airlines, the results of service evaluation of Hainan airline according to some evaluating activities are usually relatively unsatisfied. The airfares in some air routes are relatively low and part of the routes are based on the strategy of ‘low cost and low price’ to seize the market. The International airline and Southern airline usually pay more attention to passenger satisfaction, the airfare in the same air route is always higher than of Hainan airline. In the interval of low service quality level, the Hainan airline and some other airlines with the strategy of ‘low service and low airfare’ have more competitive advantages. At the high service quality level, the market shares of International aviation and Southern airline which mainly focus on service quality are significantly higher than that of Hainan airline. At the same time, at the interval of a high level of service quality, the gap between Hainan airline and International airline in the market share is much larger than the gap than appearances at the low-level service quality interval. These fully show that the improvement of service quality can effectively enhance the market competitiveness of airlines.

The values of basic market demand of Hainan airline, Southern airline, Eastern airline and International airline is respectively *a*_1_ = 5.0, *a*_2_ = 4.9, *a*_3_ = 5.1, *a*_4_ = 5.2; the service quality level is *s*_1_ = 4.52, *s*_2_ = 3.92, *s*_3_ = 3.72, *s*_4_ = 3.53; the sensitivity coefficient of service quality are *f*_1_ =1.6, *f*_2_ = 1.9, *f*_3_ = 2.0, *f*_4_ = 2.1; the joint influence coefficient of service quality are *g*_1_ = 0.4, *g*_2_ = 0.45, *g*_3_ = 0.48, *g*_4_ = 0.5; the sensitivity coefficient of airfare are *e*_1_ = 1.6, *e*_2_ = 1.2, *e*_3_ = 1.0, *e*_4_ = 0.9; the joint influence coefficient of airfare are *d*_1_ = 0.80, *d*_2_ = 0.60, *d*_3_ = 0.75. Finally, the equilibrium airfare can be obtained according to the use of MATLAB, that is $p_{1}^{*}=19.40$, $p_{2}^{*}=21.72$, $p_{3}^{*}=22.63$, $p_{4}^{*}=26.98$.

### 4.2 The impact of service quality level on airfare

**Fig 3:**
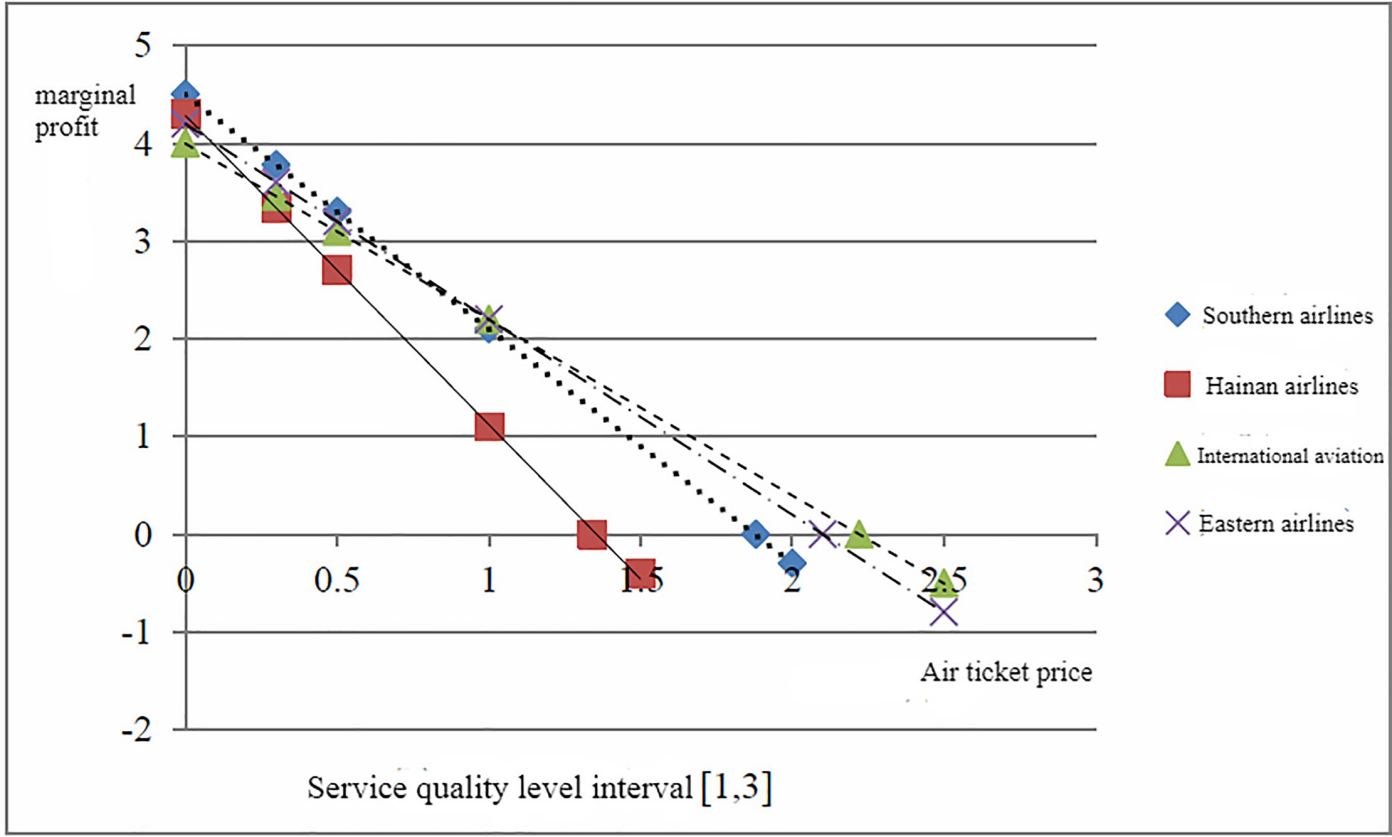
Low-level service quality level fare situation.

**Fig 4:**
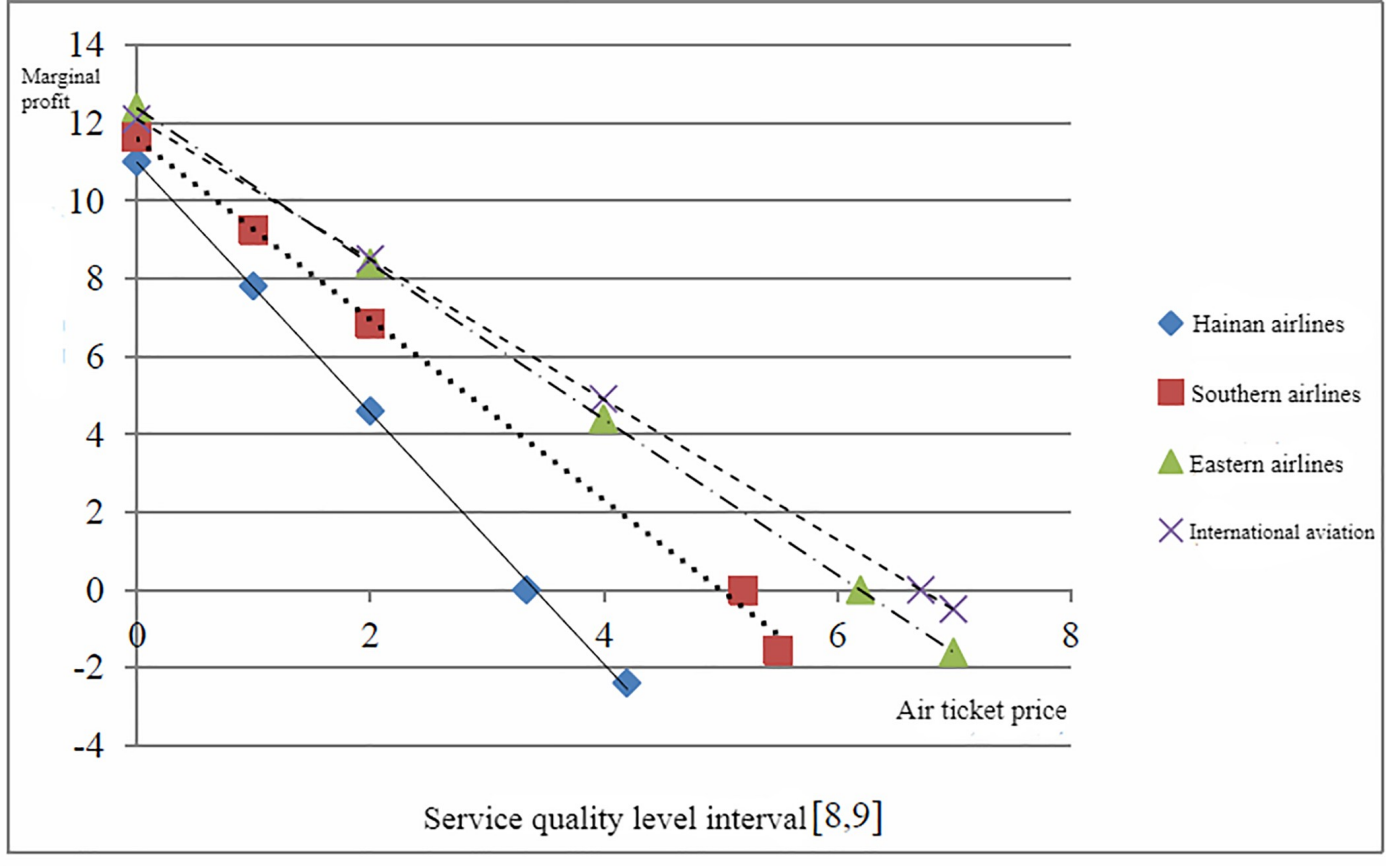
High-level service quality level fare situation.

Fig 3 shows the marginal profit of each airline changes as the airfares at the low level of service quality. Fig 4 shows the marginal profit of each airline changes as the airfare at the high level of service quality.

The vertical coordinate is the marginal profit and the horizontal coordinate is airfare. The intersection of each line and the horizontal coordinate represents the equilibrium airfare at a certain level of service quality. When the service quality level improves, the equilibrium price increases, if airlines effectively achieve the cost control, the benefits can increase with service quality levels increase. At the low level of service quality, the gap of equilibrium airfare between Hainan airline and International aviation is relatively small. At the high level of service quality, the gap of equilibrium airfare between the four airlines significantly larger. It indicates that with the increase in people's demand for trip services, the airlines which ignores the service quality and increase profits and market share only by lowering fares will be finally phased out. The airlines that focus on service quality and improve passenger satisfaction as a business strategy can achieve sustainable development. The low-cost airlines achieve long-term development, only by changing their business philosophy that focusing on the improvement of service quality and reducing the business cost.

### 4.3 Dynamic analysis

**Fig 5:**
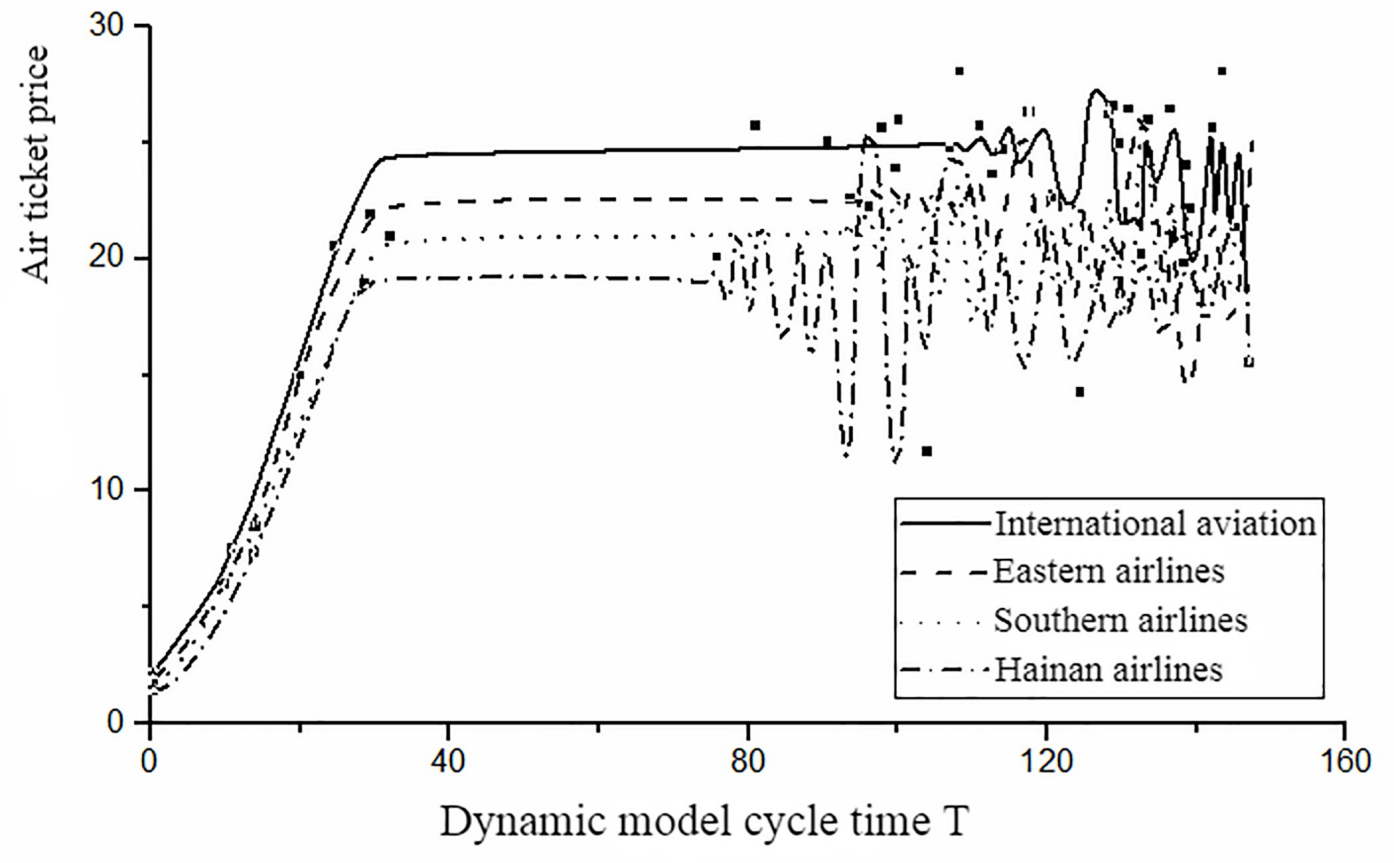
Airline price dynamic competition curve.

The dynamic model is simulated by MATLAB in this section, and the scientific and superiority of the model is verified. The growth coefficient in the variable cost is taken as *θ*_*i*_ = 0.028(*i* = 1,2,3,4); the initial two group airfares are *p*_1_ = 2.2, *p*_2_ = 2.4, *p*_3_ = 2.6, *p*_4_ = 2.8; *p*_1_ = 3.2, *p*_2_ = 3.4, *p*_3_ = 3.6, *p*_4_ = 3.8 respectively; the rate of price adjustment is specified as (*β*_*i*_+*α*_*i*_) = 0.8(*i* = 1,2,3,4);the value of the weighting factor of service quality is set as *β*_1_ = 0, *β*_2_ = 0.15, *β*_3_ = 0.6, *β*_4_ = 0.8. The model is stimulated and the equilibrium airfares are obtained, that is $p_{1}^{*}=18.38,\;p_{2}^{*}=21.79,\;p_{3}^{*}=23.69,\;p_{4}^{*}=25.40$. The process of competition is showed in Fig 5.

When T = 35, the system achieves the equilibrium state, and during the stage of [36,87] the system is in a stable. In the case of the consistency of the rate of price adjustment of each airline, the Hainan airline at T = 87 when first entering the disordered state, and the competition of International aviation become disorder at T = 101. This is because the Hainan airline does not consider the impact of service quality of the previous cycle, that is *β*_1_ = 0. When *β*_1_ = 0.8, namely International aviation fully considered the impact of the service quality of the previous cycle on the price strategy. Thus, the service quality can effectively prolong the time of system equilibrium and delay the appearance of chaotic state when it is taken as an important factor that impacting the dynamic adjustment of airfare. The chaotic state disturbs the market order and brings risks for airlines. The airlines should anticipate the disordered state and take some measures to avoid this risk.

**Fig 6:**
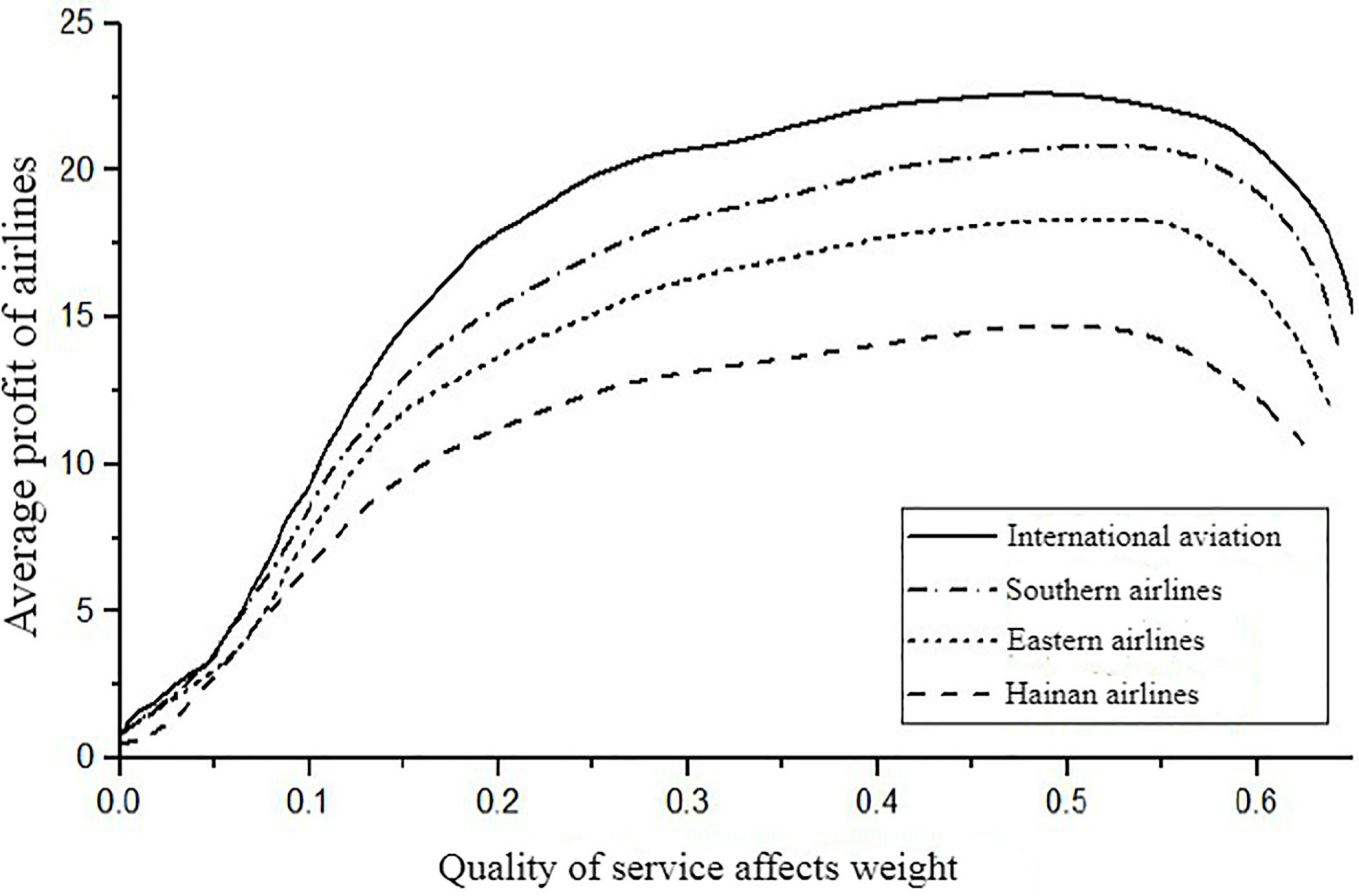
At T = 35, the effect of the *β*_4_ change on average profit.

**Fig 7:**
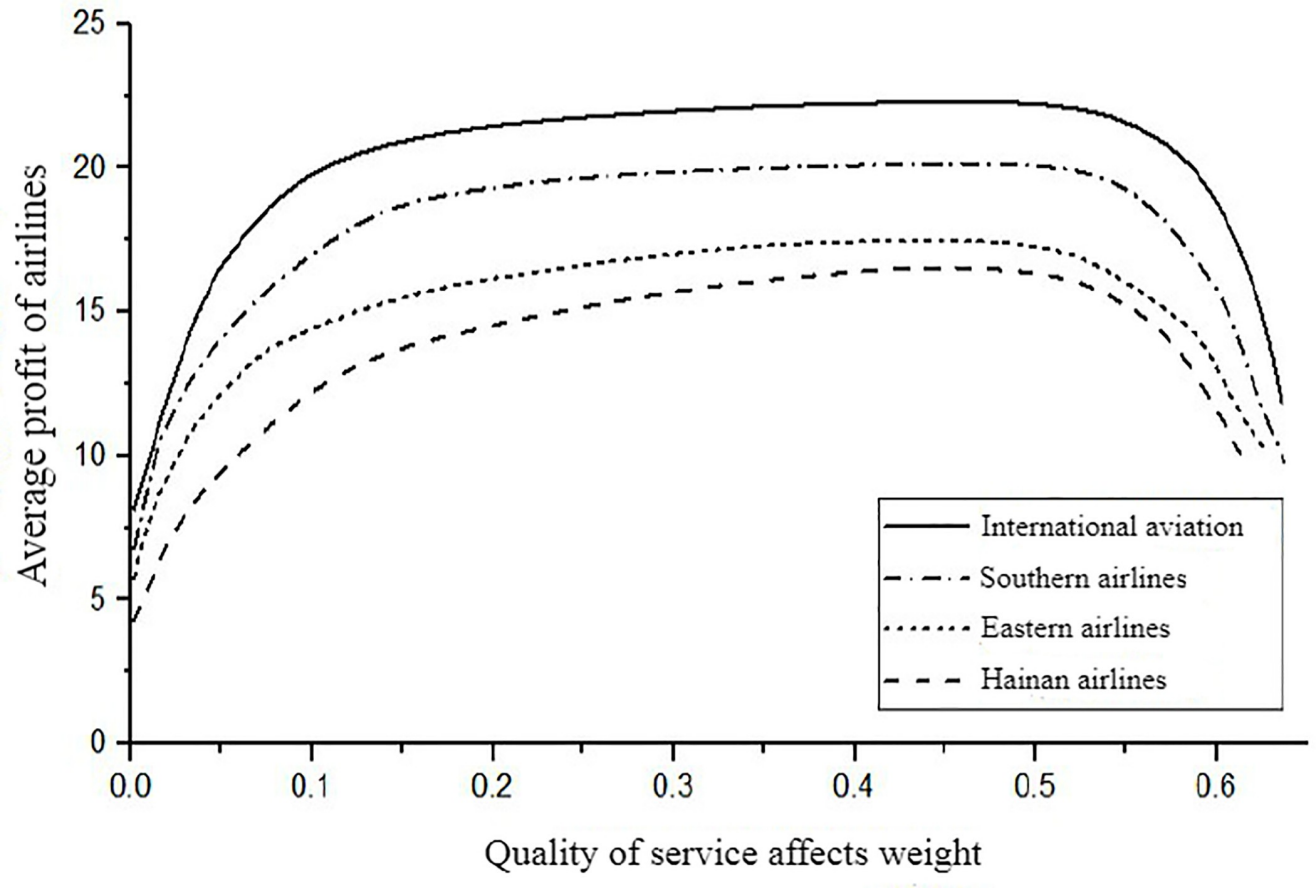
At T = 88, the effect of the *β*_4_ change on average profit.

In order to further research the impact of price adjustment speed on the profit of each oligopolistic airline when the system is stable, set the macro price adjustment speed *α*_*i*_ of each airline to be consistent, and let *α*_1_ = *α*_2_ = *α*_3_ = *α*_4_ = 0.05 and discussed separately in the early and late stages of the competition, that is while T = 35 and T = 88, focusing on the changes in the average profit of each airline with the weight of service quality *β*_*i*_. The average profit of airline *i* at the number of cycles *T* is ${\bar{\pi }}_{iT}=\sum_{i=1}^{T}\pi_{it}$. Let *β*_1_ = *β*_2_ = *β*_3_ = 0.2, and calculate the price adjustment speed of the airline in the stable domain, the value range of *β*_4_ is [0,0.5]. As shown in Fig 6, when T = 35, the average income of the four airlines continues to increase as the weight of service quality of the fourth airline *β*_4_ increases. However, as the weight *β*_4_ of the service quality exceeds a certain value, the growth rate of the average profit becomes slower. After the *β*_4_ is further increased, the average profit of each airline has a downward trend. This shows that when the adjustment speed exceeds its stability domain, the airline's profit does not rise and fall, which is unfavorable for the development of the airline. The airline should take measures to avoid this risk. When T = 88, as shown in Fig 7, the average profit of airlines increases sharply with the increase of *β*_4_, but the growth of profits will gradually slow down. This shows that in the later stage of system stability, with the increasing emphasis on service quality, airlines still have the opportunity to achieve further profitable growth.

In summary, in the early stage of the competition, the weight of the service quality impact of the airlines in time is small, and the average profit of the airlines can also generate large fluctuations. As the weight of service quality is increased, the average profit of each airline has increased significantly. When the influence weight is increased to a certain value, and the price adjustment speed is too high, the average profit of each airline is reduced, at this time, the price adjustment speed exceeds the stable area of the system, which leads to a decrease in the profit even if the air ticket price is continuously increased. At the end of the system stability domain, even if the weight of the service quality of the airline changes greatly, the ticket price increases rapidly, and the result can only cause a slight increase in the average profit. It shows that if airlines want to achieve long-term development, they should position themselves as a development strategy to seize the market with high service quality level in the early stage of route development. Because in the later stage of market competition, the improvement of service quality level and price adjustment rate can only lead to the growth of market demand, while the effect of profit growth is not obvious.

The dynamic model proposed in this paper, not only consider the influence of airfares on the market demand, but also the impact of service quality on the competition. The time required for the system to reach equilibrium is less than the tradition pricing model that only considers the price differentiation. Therefore, the dynamic pricing based on service quality proposed is more optimized and comprehensive.

## 5. Conclusion

In the context of the increasingly fierce competition in China's aviation transportation market, price strategy is still the most effective and advanced method for enterprise benefits management. However, in the current research on airline price strategy, the factor of service quality is rarely considered. With the change of market consumption structure, the service quality of enterprises has gradually become an important factor for consumers to choose.

At present, the existing research on airline revenue management mainly relies on traditional price models, such as Hotelling pricing model, differentiation-based competition model and pricing model based on general demand function. Airline pricing strategy research focuses on dynamic pricing, overbooking control, market forecasting, and cabin control. However, the factors affecting these airline pricing models are not comprehensive and do not take into account the impact of increasingly important service quality levels. It is not possible to objectively and comprehensively extract the dynamic competitive characteristics of the air transport market. Therefore, these pricing models still need to be further improved and expanded.

In response to such problems, this paper considers the impact of airline service quality and competitor service quality on pricing strategy when constructing the airline pricing model, based on the profit maximization pricing goal, and introduces the airline's service. The quality feature parameter team model was revised to construct an airline pricing model based on service quality. The quantification of the quality of service factor as a decision variable is incorporated into the pricing model, making the formulation of the airline's pricing strategy more comprehensive and reasonable. The pricing model of the multi-oligopoly airline has been established, which is more complicated than the previous model and can more accurately describe the competition status of China's air transportation market. It shows that when airlines formulate price strategies, they consider the importance of their service quality level to their sustainable development, thus guiding airlines to go further in the fierce market competition.
